# Local Over-Expression of VEGF-D^ΔNΔC^ in the Uterine Arteries of Pregnant Sheep Results in Long-Term Changes in Uterine Artery Contractility and Angiogenesis

**DOI:** 10.1371/journal.pone.0100021

**Published:** 2014-06-30

**Authors:** Vedanta Mehta, Khalil N. Abi-Nader, Panicos Shangaris, S. W. Steven Shaw, Elisa Filippi, Elizabeth Benjamin, Michael Boyd, Donald M. Peebles, John Martin, Ian Zachary, Anna L. David

**Affiliations:** 1 Institute for Women's Health, UCL, London, United Kingdom; 2 BSU, Royal Veterinary College, Camden, London, United Kingdom; 3 Centre for Cardiovascular Biology and Medicine, Division of Medicine, Rayne Building, UCL, London, United Kingdom; University of Illinois at Chicago, United States of America

## Abstract

**Background:**

The normal development of the uteroplacental circulation in pregnancy depends on angiogenic and vasodilatory factors such as vascular endothelial growth factor (VEGF). Reduced uterine artery blood flow (UABF) is a common cause of fetal growth restriction; abnormalities in angiogenic factors are implicated. Previously we showed that adenovirus (Ad)-mediated VEGF-A_165_ expression in the pregnant sheep uterine artery (UtA) increased nitric oxide synthase (NOS) expression, altered vascular reactivity and increased UABF. VEGF-D is a VEGF family member that promotes angiogenesis and vasodilatation but, in contrast to VEGF-A, does not increase vascular permeability. Here we examined the effect of Ad.VEGF-D^ΔNΔC^ vector encoding a fully processed form of VEGF-D, on the uteroplacental circulation.

**Methods:**

UtA transit-time flow probes and carotid artery catheters were implanted in mid-gestation pregnant sheep (n = 5) to measure baseline UABF and maternal haemodynamics respectively. 7–14 days later, after injection of Ad.VEGF-D^ΔNΔC^ vector (5×10^11^ particles) into one UtA and an Ad vector encoding β-galactosidase (Ad.LacZ) contralaterally, UABF was measured daily until scheduled post-mortem examination at term. UtAs were assessed for vascular reactivity, NOS expression and endothelial cell proliferation; NOS expression was studied in *ex vivo* transduced UtA endothelial cells (UAECs).

**Results:**

At 4 weeks post-injection, Ad.VEGF-D^ΔNΔC^ treated UtAs showed significantly lesser vasoconstriction (E_max_144.0 v/s 184.2, p = 0.002). There was a tendency to higher UABF in Ad.VEGF-D^ΔNΔC^ compared to Ad.LacZ transduced UtAs (50.58% v/s 26.94%, p = 0.152). There was no significant effect on maternal haemodynamics. An increased number of proliferating endothelial cells and adventitial blood vessels were observed in immunohistochemistry. Ad.VEGF-D^ΔNΔC^ expression in cultured UAECs upregulated eNOS and iNOS expression.

**Conclusions:**

Local over-expression of VEGF-D^ΔNΔC^ in the UtAs of pregnant mid-gestation sheep reduced vasoconstriction, promoted endothelial cell proliferation and showed a trend towards increased UABF. Studies in cultured UAECs indicate that VEGF-D^ΔNΔC^ may act in part through upregulation of eNOS and iNOS.

## Introduction

The normal development of the placenta is key to ensuring an uncomplicated pregnancy with adequate fetal growth. During early pregnancy increased maternal cardiac output and trophoblast driven modification of the uterine spiral arteries result in a dramatic increase in uterine perfusion [1] and a fall in utero-placental resistance, allowing provision of sufficient oxygen and nutrients for exchange across the placenta. Failure of this normal physiological process leads to fetal growth restriction (FGR) and pre-eclampsia (PET), two of the most challenging obstetric complications. Despite several pre-clinical and clinical trials of novel drugs and interventions, no effective therapies have been developed.

The fall in utero-placental resistance in normal pregnancy is mediated by interstitial extravillous trophoblast secretion of angiogenic and vasodilatory factors such as vascular endothelial growth factor (VEGF-A) to promote local blood flow to the uterus [2], [3]. VEGF induces vasodilatation and increases blood flow in diverse vascular beds [4], [5], effects mediated partly through its stimulation of endothelial production of NO [6] and prostacyclin [7]. In FGR and PET, there is decreased depth and density of trophoblast invasion of the spiral arteries [8], [9] and myometrial small arteries show increased vasoconstriction and decreased endothelium-dependent vasodilatation [10], [11]. The invading cytotrophoblasts secrete VEGF to regulate their acquisition of an endothelial-like phenotype which allows them to replace the maternal cells that line the uterine vessels. These cells also depend on VEGF for their maintenance and growth [2]. In established FGR, serum levels of VEGF-A_165_ are significantly diminished [12]. In PET, placental-derived sFlt-1, a soluble receptor of VEGF is upregulated, resulting in lowered circulating concentrations of free VEGF and endothelial dysfunction [13]. Therapeutic strategies targeting angiogenesis and vasodilatation in the uteroplacental circulation may therefore be of use in treating FGR and PET.

Previously, we have demonstrated that local over-expression of VEGF-A_165_ in the uterine arteries of pregnant sheep from mid-gestation mediated by adenovirus vector (Ad) transduction, results in a significant increase in uterine artery blood flow (UABF) for up to one month after delivery and a reduction in uterine artery contractility at term [14], [15]. These effects are associated with a short term increase in endothelial NO synthase (eNOS) in the uterine arteries (UtAs) and long term UtA adventitial neovascularization [15]. We also reported an upregulation of VEGFR-2 in the UtAs transduced with Ad.VEGF-A_165_, suggesting this may be the primary receptor mediating the biological effects observed. This was achieved without affecting maternal or fetal haemodynamic parameters.

VEGF-D^ΔNΔC^ [16] is a fully processed form of the VEGF family member, VEGF-D, generated by proteolytic processing of the N- and C-termini of full length VEGF-D, which has been shown to have a significant angiogenic and vasodilatory effect, whereas the full-length form is primarily lymphangiogenic. VEGF-D^ΔNΔC^ elicits a more restricted range of biological responses compared with VEGF-A_165_ [16], [17] including less vascular permeability. In this study we investigated the effect of Ad-mediated over-expression of VEGF-D^ΔNΔC^ in the UtAs and uterine artery endothelial cells (UAECs) of normal pregnant sheep, on eNOS levels, angiogenesis, vascular reactivity, UABF and maternal haemodynamics to determine its suitability as a therapeutic agent for FGR.

## Methods

### Ethics Statement

All work was conducted in accordance with the UK Animals (Scientific Procedures) Act (1986), project licence 70/6546 and approved by the Royal Veterinary College ethics committee and the UCL Biological Services Unit ethics committee.

### Animals

All experiments were carried out in time-mated normal pregnant sheep (Romney breed), not affected by vascular placental insufficiency. Mid-gestation pregnant sheep (n = 6, 82–109 days of gestation, term = 145 days) were studied 4–7 days after vector administration, (“short term”) for organ bath experiments to examine vascular reactivity, eNOS activity, neovascularization and to assess acute toxicity (if any). A separate “long term” group of mid-gestation pregnant ewes carrying singleton (n = 4) or twin pregnancies (n = 1) (82–98 days of gestation) were studied until the end of gestation (Table 1) for assessment of eNOS activity, neovascularization, UABF measurements, and maternal haemodynamics. In addition, two sheep were injected with only the vehicle (PBS) and sacrificed at the short-term time point and long-term time-point each (after injection), to provide control tissue for histological and haematologic analysis. Normal mid-gestation pregnant sheep (n = 6, 90–100 days) were used to provide uterine artery endothelial cells (UAECs) for experiments.

**Table 1:** Analysis performed on experimental sheep and long-term changes in UABF from baseline to 28 days after vector injection.

Animal	Fetal number	Post mortem examination (d after vector injection)	Side of vector injection		% Change in UABF at 28 days	
			Ad.VEGF-D^ΔNΔC^	Ad.LacZ	Ad.VEGF-D^ΔNΔC^	Ad.LacZ
1	Singleton	4	Gravid	Non-gravid	NA	NA
2	Singleton	6	Gravid	Non-gravid	NA	NA
3	Singleton	7	Non-gravid	Gravid	NA	NA
4	Singleton	7	Gravid	Non-gravid	NA	NA
5	Singleton	5	Non-gravid	Gravid	NA	NA
6	Singleton	4	Non-gravid	Gravid	NA	NA
7	Twin	43	Gravid	Gravid	23.60	21.23
8	Singleton	41	Non-gravid	Gravid	66.14	47.86
9	Singleton	30	Gravid	Non-gravid	24.77	4.48
10	Singleton	34	Non-gravid	Gravid	32.65	19.85
11	Singleton	45	Non-gravid	Gravid	105.76	41.32

UABF: Uterine artery blood flow; VEGF: Vascular Endothelial Growth Factor; NA: Not available; d: days.

Animals 1–6 were used for short-term experiments.

Animals 7–11 were used for long-term experiments, involving chronic implantation of telemetric flow probes around the uterine arteries.

### Animal surgery and vector injection

After fasting overnight, pregnant ewes at 90.60±6.19 days of gestation underwent general anaesthesia induced with thiopental sodium 20 mg/kg IV (Thiovet, Novartis Animal Health UK Ltd, Hertfordshire, UK) and maintained with 2–2.5% isoflurane in oxygen (Isoflurane-Vet, Merial Animal Health Ltd, Essex, UK) after intubation. Umbilical artery Doppler measurements, pulsatility index (PI) and resistance index (RI) were acquired [14]. Gestational ages were confirmed by ultrasound using fetal measurements [18]. For chronic maternal haemodynamic monitoring (n = 4), a blood pressure sensitive PA-D70 catheter (Data Sciences International, Tilburg, Netherlands) was inserted into the carotid artery lumen, as described [15]. A laparotomy was then performed, the UtAs were identified bilaterally and mobilised immediately proximal to the first bifurcation. For long term experiments, a transit time flow probe (6 mm 6PS, Transonic Systems Inc., NY, USA), which can measure blood flow with an absolute accuracy of ±10% was placed around the main UtA on each side. The cabling from each probe was then exteriorized onto the ewe's right flank, and the skin buttons were secured to the skin as described [19]. The abdomen was closed, the ewe received standard analgesia and antibiotic prophylaxis and the animal was then recovered.

For long term experiments, vector was delivered to the UtAs 7–14 days after flow probe placement (at 100.6±7.63 days of gestation). For short-term experiments, sheep only had the vector injection surgery at 97.5±12.72 days of gestation without any prior probe placement surgery. To deliver the Ad vectors into the UtAs, the sheep underwent a second general anaesthetic and laparotomy. The UtAs were identified bilaterally and mobilised immediately proximal to the first bifurcation, or proximal to the position of the flow probes. A butterfly needle (21 Gauge) was inserted into the UtA and the viral vector (5×10^11^ viral particles in 10 ml phosphate buffered saline) was injected over a 1 minute period, during which time the UtA was digitally occluded proximal to the site of injection and for a further 4 minutes after removal of the needle [14]. The operators were blinded to which horn of the uterus received Ad.VEGF-D^ΔNΔC^ at the time of vector injection. Ad.LacZ vector (5×10^11^ viral particles in 10 ml phosphate buffered saline) was injected into the contralateral UtA. The ewe received standard analgesia and antibiotic prophylaxis, and the abdominal incision was closed [19].

#### Animal monitoring

Measurements of UABF, maternal blood pressure and heart rate were recorded continuously in the instrumented ewes, over the 3 days preceding and 7 days succeeding vector injection to capture acute effects, and for 1 hour daily thereafter, at the same hour of the day, to capture chronic effects.

UABF was sampled at a rate of 128 Hz and data were transmitted telemetrically via the skin buttons when they were connected to the PhysioGear I transmitter system and to the PhysioView Data Acquisition Software (Transonic Systems Inc.). The data acquired from the flow probes were analyzed using Acknowledge software 3.9.1 (Biopac Q10 Systems Inc., CA, USA). The baseline UABF was calculated as the average of three daily mean UABF measurements taken for one hour each day before vector injection. UABF percentage change from baseline was calculated at specified time points, 7, 14, 21 and 28 days after vector injection, using the average of three consecutive daily mean UABF measurements on the day of and one day either side of the time point. A two-way General Linear Model (GLiM) was used to compare the UABF percentage change in Ad.VEGF-D^ΔNΔC^ and Ad.LacZ-injected UtAs at each time point and also the gradients of UABF percentage change over the length of gestation. The two factors accounted for in the GLiM analysis were whether the UtA supplied a gravid or non-gravid horn and whether Ad.VEGF-D^ΔNΔC^ or Ad.LacZ vector was injected.

Maternal BP and HR were recorded telemetrically. Uploaded traces were analyzed using Dataquest ART 4.1 software (Data Sciences International). A two-tailed paired t-test was used to compare changes in BP before and after the administration of the vector.

#### Tissue sampling

Terminal anaesthesia was performed either one week after vector administration (in short-term animals) or at the end of gestation (136 to 142 days) to allow sampling of the UtAs under optimal conditions. The umbilical artery PI and RI were measured using ultrasound Doppler and compared with pre-injection values. The UtAs and their next three divisions down to the level of the uterine wall (vessel diameter 1 mm) as well as other maternal and fetal tissues were sampled as described previously [14]. Tissue samples to be used in histological and immunohistochemical studies were fixed in 4% paraformaldehyde for 24 h and then transferred into 70% alcohol to be subsequently blocked in paraffin. All other samples were snap frozen in liquid nitrogen and stored at −80°C.

#### Organ bath studies for UtA reactivity

The cleared second and third UtA branches were divided into 3 mm long segments and examined on two 8-chambered organ bath setups in the absence and presence of inhibitors as described, namely L-NAME (300 µM), an NO synthase inhibitor; NS-398 (10 µM), a cyclooxygenase inhibitor and Apamin (1 µM) a blocker of SK channels that inhibits the actions of endothelium-derived hyperpolarizing factor [15].

#### Histological and blood examination

Paraffinized tissue sections stained with hematoxylin and eosin were observed microscopically for histological examination. Maternal and fetal blood samples obtained before vector injection and at post mortem (from both the short-term and long-term cohorts) were tested for routine haematology, biochemistry and liver function tests at the Clinical Diagnostics Laboratory, RVC Hawkeshead.

#### Assessment of endothelial cell proliferation and neovascularization

Paraffinised sections of the main UtA were double stained with anti-BrdU and anti-vWF antibodies to assess endothelial cell proliferation and adventitial neovascularization, respectively. Tissue sections were dewaxed and endogenous peroxidase activity was blocked with 0.6% Hydrogen peroxide for 15 minutes. Antigen retrieval was performed using 0.1% trypsin (215240, BD Biosciences, UK) digestion at 37°C for 10 minutes. The sections were then blocked with 5% non-immune donkey serum (D9663, Sigma Aldrich, Gillingham, Dorset, UK) at room temperature for 30 minutes. Polyclonal rabbit anti-human vWF (1∶400, A0082, Dako, Glostrup, Denmark) was used as the primary antibody and incubated overnight at 4°C, followed by a biotinylated donkey anti-rabbit secondary antibody (1∶100, 711-065-152, Jackson ImmunoResearch, West Grove, PA, USA) for 1 hour at room temperature. Following one wash with PBS supplemented with 0.1% bovine serum albumin and two washes with PBS, the sections were incubated with ABC solution (PK4000, Vector Laboratories, Peterborough, UK) for one hour. The deposited ABC complex was detected via covalent conjugation of biotinylated tyramide (1∶1000, Dupont, UK), reacted in the presence of 0.01% Hydrogen peroxide in PBS for 10 minutes at room temperature [20]. This treatment with biotinylated tyramide allowed us to transform an initially noncovalent form of biotin labeling into a covalent one, to allow the label to withstand the post-treatment with 2N Hydrochloric acid (HCl) for 45 minutes at room temperature needed to expose the BrdU antigen. The HCl-treated sections were briefly washed in PBS, exposed for 30 minutes to 0.1M boric acid/sodium borate buffer (pH 8.5), washed in PBS again, pre-incubated with 0.5% Triton X-100 and 5% donkey serum in PBS and then incubated with the primary mouse antibody against BrdU (1∶100 Dako, Glostrup, Germany M0744) overnight at 4°C. This step was followed by a one hour incubation with a secondary Alexafluor-488 conjugated goat anti-mouse IgG (1∶200, 11001, Invitrogen, Paisley, UK) and then enhanced with a tertiary Alexafluor-488 conjugated donkey anti-goat antibody (1∶200, 11055, Invitrogen, Paisley, UK), in combination with Texas Red Streptavidin (016-070-084, Jackson Immunoresearch) for one hour at room temperature. The sections were covered with mounting medium with DAPI (H-1200, Vector Laboratories, UK) and stored in the dark at 4°C before use. Negative controls were obtained by not exposing the tissue section to either of the primary antibodies.

#### Confocal Microscopy

For visualizing the immuno-fluorescence double labeling, digital micrographs of the Alexafluor-488 for the BrdU staining and Texas red fluorescence for vWF were taken representing an area of 1 mm×1 mm (1024 pixels×1024 pixels; grayscale 0–255) with a Leica TCS 4D confocal laser microscope using a 20× objective (Milton Keynes, UK). The fluorescence was excited using low ArKr laser power (0.25 V) at wavelengths of 488 nm for Alexafluor-488, 568 nm for Texas Red and 358 nm (ultraviolet) for DAPI, and detected using the BP-FITC filter for Alexafluor-488, the LP590 filter for Texas Red and the LP360 filter for DAPI, respectively. Nine consecutive, equidistant levels were recorded and condensed to a single bitmap using the MaxIntens algorithm. Proliferating endothelial cells and adventitial blood vessels (with a distinct lumen) were identified and counted by two independent observers who were blinded to the treatment. All analysis was performed in duplicate.

#### Measurement of VEGF-D protein expression

The quantity of human VEGF-D protein in snap frozen samples of UtA, uterine wall and whole placentome from two pregnant sheep in the short-term study and long-term study each was measured by enzyme-linked immunosorbent assay (R&D Systems, Minneapolis, MN, USA) as described previously [14]. Human VEGF-D levels were also measured in pre- and post-injection maternal serum samples and fetal serum samples collected at post-mortem examination.

#### Measurements of phosphorylated and total eNOS, Akt and Erk levels

Protein extracts from the snap-frozen UtA tissues from both the short-term and long-term studies were used to estimate levels of phosphorylated(p)-eNOS(Ser^1177^, 1∶1000, 9570, Cell Signaling Technology, Danvers, MA, USA), total (T)-eNOS (1∶3000, 610296, BD Transduction Laboratories), p-Akt (Ser^473^, 1∶1000, 9271, Cell Signaling Technology), T-Akt (1∶1000, 4691, Cell Signaling Technology), p-Erk (Thr^202^/Tyr^204^, 1∶1000, 9101, Cell Signaling Technology) and T-Erk (1∶1000, 9102, Cell Signaling Technology) by western blotting, as previously described [15].

#### Uterine artery endothelial cell (UAEC) isolation

UtAs from normal mid-gestation pregnant sheep (approximately 90–100 days, n = 6) were dissected free of surrounding connective tissue and cleared from their origin at the internal iliac artery up to the level of the 2nd division, under terminal anaesthesia, as described above. The ewe was then put down with an overdose of intravenous pentobarbitone and the uterine arteries were ligated at both ends using 1-0 silk ties and removed as a single piece (which included the main, first and second branches).

The harvested UtAs were placed in a 10 cm petri-dish in a sterile laminar flow hood and cleared further of surrounding connective tissue and blood clots.

At the proximal end, a 23 gauge butterfly needle was introduced and secured tightly with a haemostat. The vessel was flushed with M-199 (50 ml, 41150-020, Invitrogen, Paisley, UK) to remove all blood clots. The distal end of the vessel was then tied with a silk tie and the vessel was inflated with Endothelial Cell Basal Medium (EBM, CC3121, Lonza, Slough, UK) containing 5 mg/ml collagenase (11088815001, Roche Diagnostics, Mannheim, Germany) and 0.5% bovine serum albumin (BSA) (A4503, Sigma Aldrich, UK) to dissociate endothelial cells from the vessel wall. The inflated vessel was incubated at 37°C for 15 minutes. The distal tie was then cut and the contents of the vessel were flushed into a falcon tube using Endothelial Cell Growth Medium (EGM, CC4133, Lonza). The endothelial cell fraction was concentrated by centrifugation and washed two times with EGM to remove all debris. The freshly isolated cells were considered to be at passage 0 and plated in 4 wells of a 6-well plate (140675, Nunc, Roskilde, Denmark) in EGM containing 10% Fetal bovine serum, 1% penicillin-streptomycin (15140-122, Invitrogen, Paisley, UK). All cell surfaces on which endothelial cells were cultured were treated with gelatin (G1393, Sigma Aldrich) to enhance adhesion to the surface. Cells were grown for approximately 6 days and passaged (passage 1) to T-25 flasks (136196, Nunc). Cells were grown to 70% confluence in T-25 flasks and then passaged (passage 2) to T-75 flasks (178905, Nunc). Cells were again grown to approximately 70% confluence and passaged once more (passage 3) to T-175 flasks (178883, Nunc). Once ready for passage, the cells were passaged (passage 4) to 6-well plates for adenovirus infection experiments. To verify their endothelial identity, primary UAECs were incubated with Ac-LDL tagged with Alexafluor-488 (L-23380, Invitrogen, UK). Ac-LDL was added directly to cells growing in culture in 1 ml EBM (serum-free) to yield a final concentration of 10 µg/ml and left to incubate for four hours at 37°C. The medium was then aspirated and fresh PBS was added. Cells were then observed under a fluorescent microscope and photographed on a confocal microscope.

#### Immunofluorescent staining of UAECs

UAECs (3.5×10^5^ cells/well) were seeded on a gelatinized cover-slip in a 6-well plate and grown to 100% confluence overnight. The next morning, the medium was aspirated and 4% formaldehyde was added gently along the edge of each well to fix the cells. The plate was shaken gently for 15 minutes, after which the formaldehyde was discarded and the cells were washed twice with PBS. 0.1% Triton X-100 (diluted in PBS) was added to each well to permeabilize the cell membrane. The solution was aspirated after 10 minutes and the cells were washed twice with PBS. Primary antibodies were prepared in PBS containing 0.1% Tween-20 and 1% BSA. The antibodies used are outlined in Table 2. After the addition of the primary antibody, the plate was left overnight at 4°C on the shaking platform. Next morning, the cells were washed three times in PBS. The appropriate secondary antibodies (Table 2) were prepared in the same solution and added to the cells for one hour at room temperature. The wells were again washed three times with PBS. The coverslip was then gently lifted up and inverted over a drop of 4′,6-diamidino-2-phenylindole (DAPI) solution on a glass slide (with the cell adherent surface of the coverslip facing down). After five minutes, the slides were observed under a fluorescent microscope and subsequently photographed on a confocal microscope. Negative controls were obtained by omission of the primary antibody.

**Table 2:** Antibodies used to confirm identity of endothelial cells isolated from pregnant sheep uterine arteries.

Primary Antibody	Secondary Antibody
polyclonal rabbit anti-human vWF (1∶400, A0082, Dako, Glostrup, Germany)	Alexafluor-488 donkey anti-rabbit IgG (1∶1000, A21206, Invitrogen, Paisley, UK)
rabbit monoclonal anti- β-catenin (1∶2000, C2206, Sigma Aldrich, Gillingham, Dorset, UK)	Alexafluor-488 donkey anti-rabbit IgG (1∶1000, A21206, Invitrogen, Paisley, UK)
mouse monoclonal anti-VE cadherin (1∶500, sc9989, Santa Cruz Biotechnology, Heidelberg, Germany)	Alexafluor-488 goat anti-mouse IgG(1∶1000, A11001, Invitrogen, Paisley,UK)

#### Infection of UAECs with Adenovirus vectors

Cultured UAECs were seeded in a six well plate (3.5×10^5^ cells/well), and infected the following day with Ad.VEGF-D^ΔNΔC^ or Ad.LacZ at multiplicities of infection (MOI) of 0, 1, 10, 100, 1000 and 10000. At the same time, serum concentration in the culture medium was changed to 0.5%. Protein was extracted after 48 hours of infection for analysis by western blotting for p-eNOS, T-eNOS, p-Akt, T-Akt, p-Erk and T-Erk as described above.

## Results

There were no cases of maternal or fetal mortality and morbidity. UABF and maternal haemodynamics were measured successfully in all the ewes with chronically implanted UtA flow probes and carotid artery blood-pressure sensitive catheters. Gross examination at the time of post mortem and microscopic histological examination of ewes and fetuses did not reveal any pathology. The UtAs did not show any evidence of edema, leucocyte infiltration or inflammation. There were no detectable changes in haematological and biochemical profiles or liver enzyme function when compared with baseline analysis in the mother at 1 week (n = 3) or 5 weeks (n = 3) after vector injection, or in the fetal sheep after vector injection when compared with controls (which had only been injected with the vehicle).

### Fetal Weights

Fetal weights from singleton pregnancies undergoing long-term UtA blood flow monitoring (n = 4) were measured at post-mortem examination and compared to a historical singleton fetal control group from the same sheep breed (n = 9). The mean gestational age of the two groups was not statistically different (139.3±2.5 days v/s 137.8±3.9 days, p = 0.97, unpaired t-test). The mean fetal weight in the experimental group was not significantly different than that in the control group (4863±492 grams v/s 4698±1004 grams, p = 0.45, unpaired t-test).

Fetal liver weights from the experimental group (n = 4) were compared to a historical singleton fetal control group from the same sheep breed (n = 10). The mean gestational age of the two groups was not statistically different (139.3±2.5 days v/s 138.9±6.5 days, p = 0.68, unpaired t-test). Mean fetal liver weight was higher in the experimental group (123.60±24.67 grams v/s 106.10±21.18 grams), although this increase was not significant (p = 0.20).

### Umbilical artery Doppler examination

Umbilical artery Doppler pulsatility index was measured at mid-gestation (before vector injection) and at term (4–6 weeks after vector injection) in fetal sheep in the uterine horn that received Ad.VEGF-D^ΔNΔC^ injection (n = 5) or Ad.LacZ injection (n = 5) or phosphate- buffered saline (PBS; n = 2). There were no significant differences in the change in pulsatility index with gestation between any of the groups examined.

### Vascular reactivity

**Figure 1:**
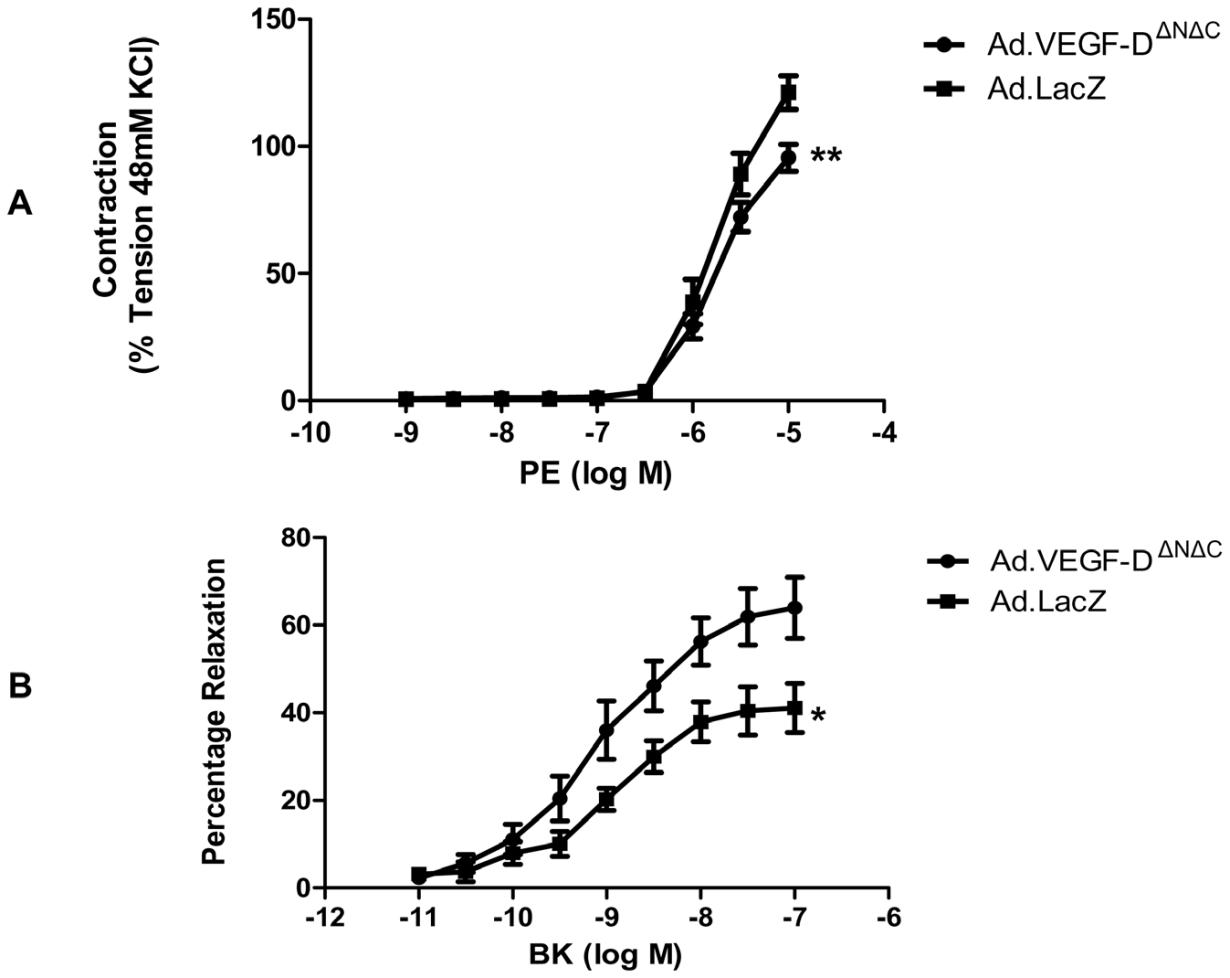
Vascular reactivity of uterine arteries 4–7 days after vector administration. (A) Logarithmic dose-response curve to L-phenylephrine (PE) depicting that the contractile tension generated in the UtAs of pregnant sheep (n = 6) is significantly lower in Ad.VEGF-D^ΔNΔC^ transduced vessels relative to Ad.LacZ transduced vessels 4–7 days post-vector injection. The contractility of the vessel is expressed as a percentage of the response to KCl. ** p<0.005. (B) Logarithmic dose-response curve to Bradykinin (BK) depicting that the relaxation response generated in the UtAs of pregnant sheep (n = 5) is significantly greater in the Ad.VEGF-D^ΔNΔC^ transduced arteries compared to Ad.LacZ treated vessels 4–7 days post-vector injection. The relaxation is expressed as a percentage of inhibition of PE-induced contractions. * denotes p = 0.05. Error bars denote SEM.

Organ bath experiments on UtA segments 4–7 days after injection showed that, compared with Ad.LacZ vessels, there was a significantly reduced mean contractile response to phenylephrine (E_max_ 126.6±7.54% v/s 159.9±10.96%, n = 6, p = 0.0001) and an increased mean relaxation response to bradykinin in Ad.VEGF-D^ΔNΔC^ transduced vessels (E_max_ 62.50±3.25% v/s 41.89±2.49%, n = 5, p = 0.05, Figure 1).

**Figure 2:**
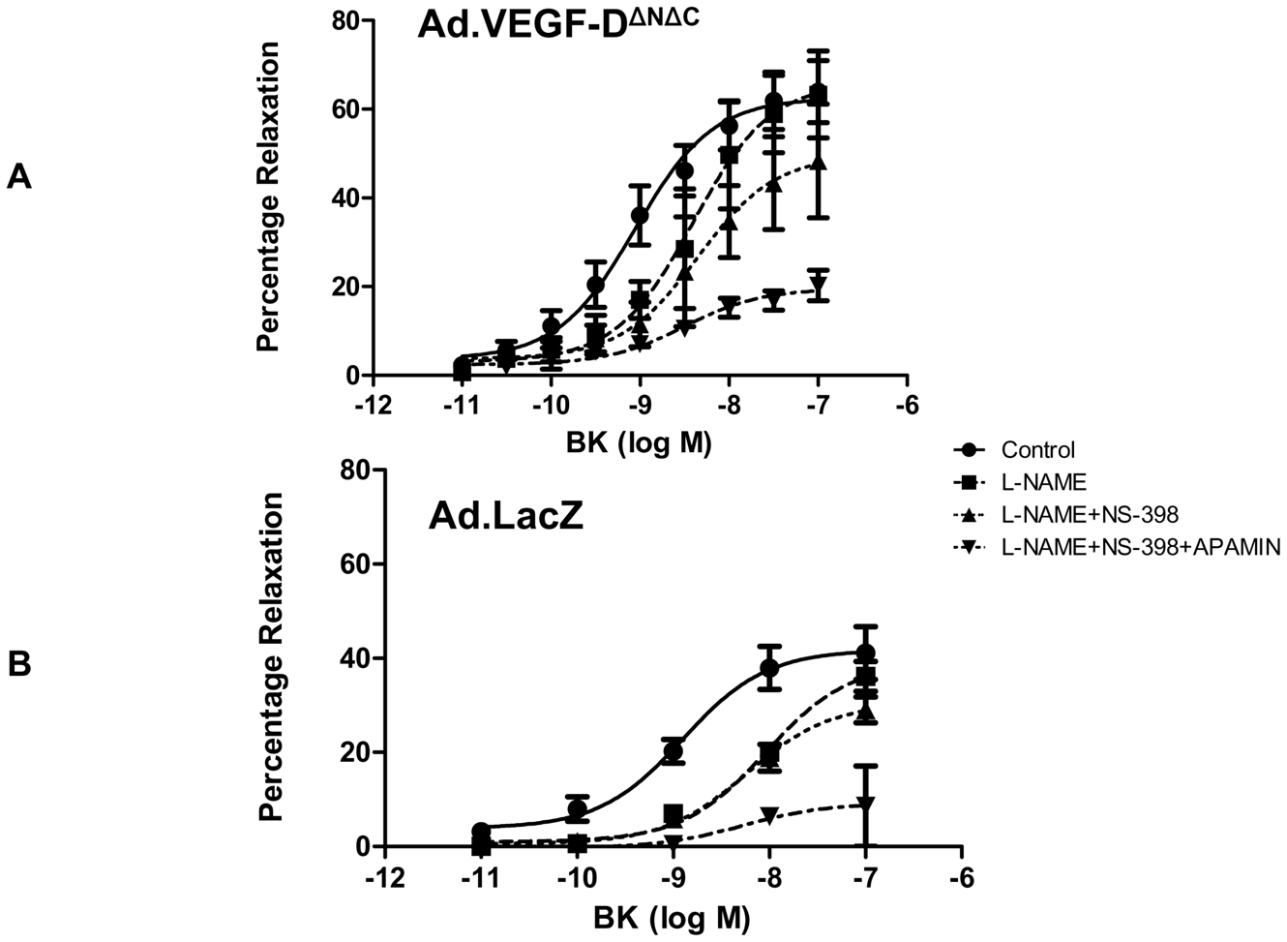
The endothelium-dependent relaxation to bradykinin in the presence of different inhibitors of the relaxation pathway in pregnant sheep uterine arteries, 4–7 days after Ad.VEGF-D^ΔNΔC^ or Ad.LacZ transduction. The contribution of NO, PGI_2_ and EDHF on the relaxation response to BK were investigated in vessels pre-contracted with PE. Cumulative relaxation curves of BK (10^−11^M to 10^−6^M) were constructed under the following conditions: (1) control (no inhibitors); (2) in the presence of L-NAME (300 µM); (3) in the presence of L-NAME and NS-398 (COX-2 inhibitor, 10 µM); (4) in the presence of L-NAME, NS-398 and apamin (1 µM). Relaxation was expressed as a percentage of inhibition of PE-induced contraction. The mean relaxation response of vessels from singleton pregnant sheep was calculated (n = 5). Statistical significance was assumed at p<0.05. The BK relaxant effect was reduced by L-NAME (p<0.05, n = 5), but not significantly modified by the further addition of NS-398. The remaining endothelium-dependent relaxation (E_max_), that was resistant to NS-398 and NO synthase inhibition, was significantly reduced by pretreatment with apamin in both Ad.VEGF-D^ΔNΔC^ and Ad.LacZ treated arteries (P<0.05, n = 5). The residual relaxation that was resistant to the cumulative addition of all three inhibitors was significantly greater in the Ad.VEGF-D^ΔNΔC^ transduced segments compared to Ad.LacZ transduced segments.

Treatment with L-NAME significantly reduced the relaxant effect of BK in UtA segments from both the Ad.VEGF-D^ΔNΔC^ and Ad.LacZ transduced vessels. Even though the E_max_ values in the presence of L-NAME were not significantly different from that of vessels unexposed to this inhibitor, addition of L-NAME resulted in a significant shift of the dose-response curve to the right (Figure 2). Further addition of NS-398 (with L-NAME) did not result in any significant change in the endothelium-dependent relaxation, even though there was a trend towards a reduction in the relaxation response in the Ad.VEGF-D^ΔNΔC^ transduced segments. Pre-treatment with Apamin (in the presence of L-NAME and NS-398) resulted in a further significant attenuation of the endothelium-dependent relaxation (Figure 2). The residual relaxation that was resistant to the cumulative inhibition of all three inhibitors was significantly greater in the Ad.VEGF-D^ΔNΔC^ transduced vessels (19.75%) compared to Ad.LacZ transduced vessels (9.21%, n = 5, p<0.05, Two-way ANOVA). NS-398 and Apamin alone had no significant influence on relaxation (data not shown).

**Figure 3:**
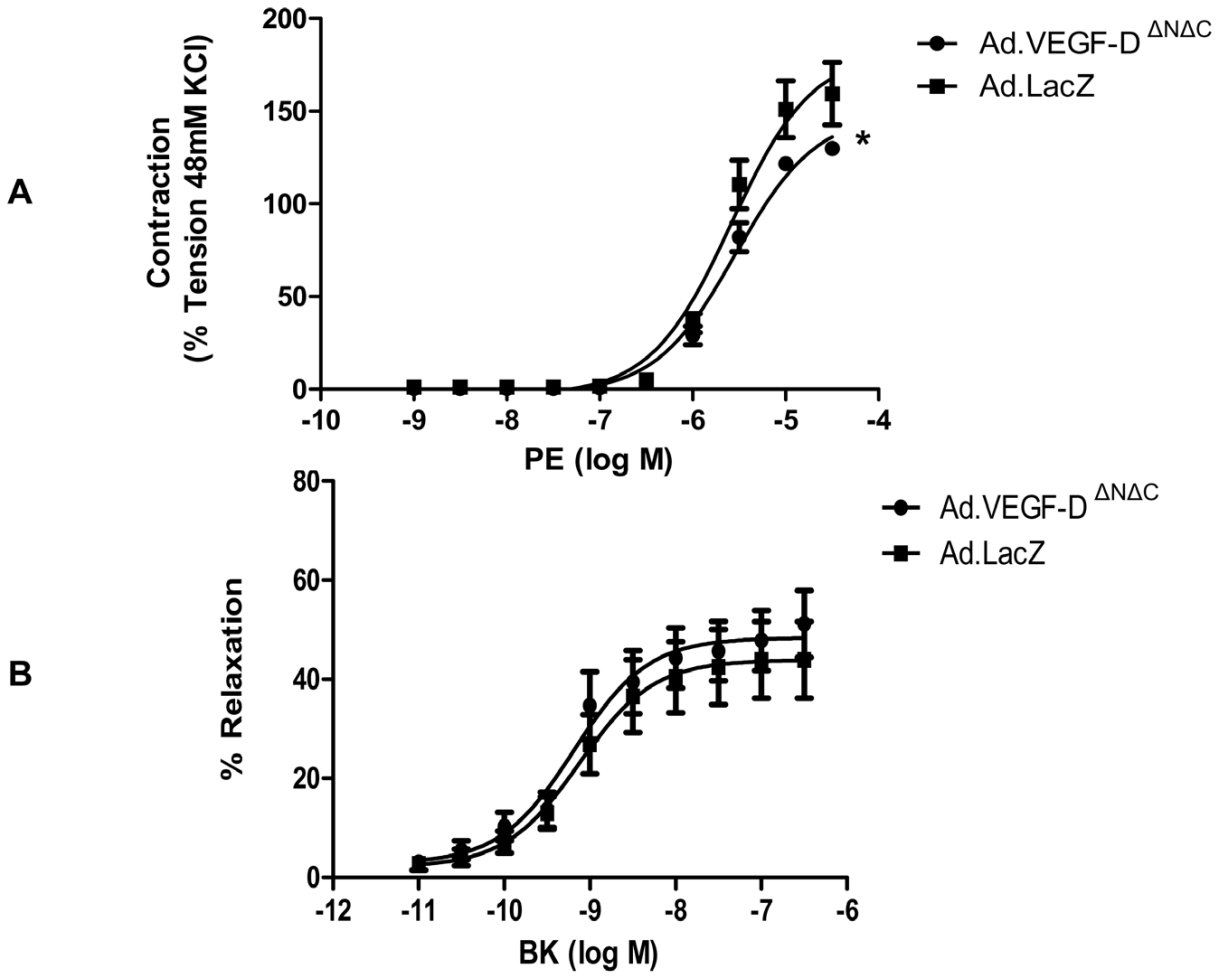
Vascular reactivity of uterine arteries 30–45 days after vector administration. (A) Logarithmic dose-response curve to L-phenylephrine (PE) depicting that the contractile tension generated in the UtAs of term pregnant sheep (n = 5) is significantly lower in Ad.VEGF-D^ΔNΔC^ transduced vessels relative to Ad.LacZ transduced vessels 30–45 days post-injection. The contractility of the vessel is expressed as a percentage of the response to KCl. * p<0.005. (B) Logarithmic dose-response curve to Bradykinin (BK) depicting that the relaxation response generated in the UtAs of term pregnant sheep (n = 5) is not significantly different between the Ad.VEGF-D^ΔNΔC^ and Ad.LacZ treated vessels 30–45 days post-injection. The relaxation is expressed as a percentage of inhibition of PE-induced contractions. Error bars denote SEM.

Organ bath experiments on UtA segments 30–45 days post vector injection showed a significantly reduced mean contractile response to phenylephrine in Ad.VEGF-D^ΔNΔC^ transduced vessels when compared with Ad.LacZ transduced vessels (E_max_ 144.0±4.64 v/s 184.2±8.58, n = 5, p = 0.002). However, we observed no significant difference in the relaxation response to bradykinin between the Ad.VEGF-D^ΔNΔC^ and Ad.LacZ transduced sides at the long-term time point (Figure 3). There was no difference in the relaxation response between the Ad.VEGF-D^ΔNΔC^ and Ad.LacZ transduced sides in the presence of the inhibitors described above (data not shown).

### The long-term effects of Ad.VEGF-D^ΔNΔC^ on UABF

UABF was measured long-term in five pregnant ewes which received UtA injection of Ad.VEGF-D^ΔNΔC^ and Ad.LacZ contra-laterally. Telemetric flow probes were implanted around the UtAs of these sheep 7–14 days before vector injection and UABF was measured for 1 hour each day at the same time of the day to avoid diurnal variation. Before the administration of the vector, the measured UABF was averaged over three consecutive days to derive a baseline value. The daily measurements of blood flow post-injection for each uterine artery were compared with this baseline value and converted into a percentage increase from baseline.

**Figure 4:**
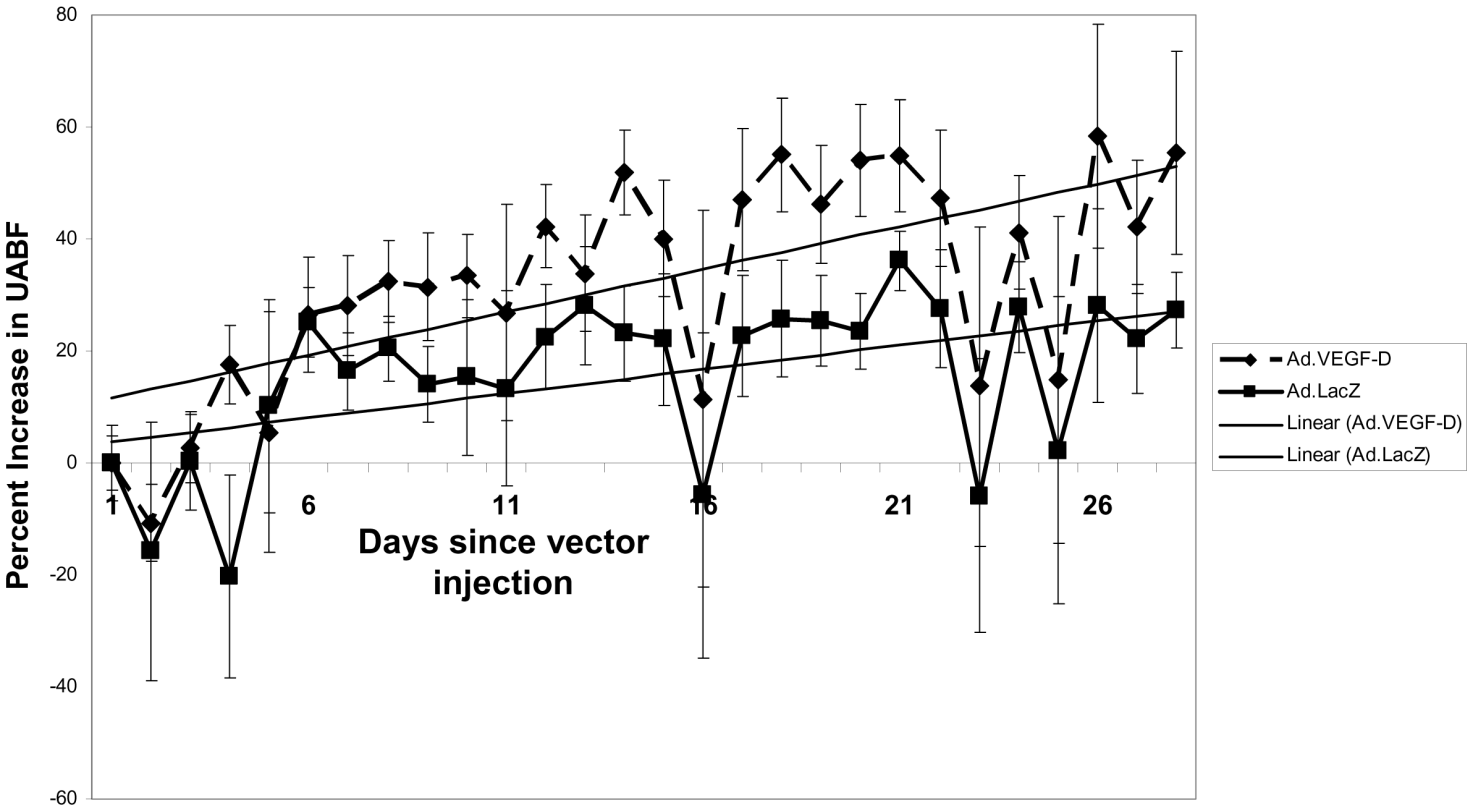
Changes in UABF after adenovirus vector injection. Graph showing the percentage increase in UABF from baseline (adjusted to 0) and gradients of percentage increase in UABF in Ad.VEGF-D^ΔNΔC^ and Ad.LacZ transduced UtAs from 5 pregnant sheep. Vector injection = Day 0; Error bars denote SEM.

As was seen in the previous study using Ad.VEGF-A_165_ injection [15], there was a slight fall in UABF from baseline for the first 1–3 days after vector injection, but it had recovered completely by day 4 in all cases (Figure 4). The mean percentage fall in UABF from baseline 1–3 days after vector injection was not significantly different in Ad.VEGF-D^ΔNΔC^ (n = 5) compared with Ad.LacZ (n = 5) injected uterine arteries (9.01±5.95% v/s 9.14±6.50%, p = 0.99).

At 28 days post vector injection, the mean increase in blood flow in the UtAs injected with Ad.VEGF-D^ΔNΔC^ tended to be higher when compared with UtAs injected with Ad.LacZ vector (50.58±15.81% v/s 26.94±7.84%, p = 0.152, n = 5, General Linear Model, Figure 4) but this difference was not significant. The mean gradient of percentage increase in UABF, defined as the slope of the percentage increase in UABF with respect to time, tended to be higher in the Ad.VEGF-D^ΔNΔC^ transduced vessels at all time points examined, that is, 7, 14, 21 and 28 days after vector injection (Table 3).

**Table 3:** Percentage change in UABF and gradient of percentage change in UABF at 1-week intervals post Ad.VEGF-D^ΔNΔC^/Ad.LacZ injection to the UtAs of pregnant sheep (n = 5).

Time-point after vector injection	% Increase in UABF ± SEM		p value (GLiM)	Gradient of % increase in UABF		p value (GLiM)
	Ad.VEGF-D^ΔNΔC^	Ad.LacZ		Ad.VEGF-D^ΔNΔC^	Ad.LacZ	
7 days	28.86±8.23	20.91±5.28	0.496	3.32	0.99	0.145
14 days	41.93±8.70	24.41±10.04	0.223	3.25	1.68	0.224
21 days	52.09±9.83	29.11±6.52	0.102	2.70	1.34	0.093
28 days	50.58±15.81	26.94±7.84	0.152	2.05	1.00	0.058

GLiM: General Linear Model.

### VEGF-D Expression

Table 4 summarizes the VEGF-D protein levels in the UtA, uterine wall and placentome samples examined from the short-term experiments as determined by ELISA. Even though not all the examined branches had detectable levels of protein in this assay, there was no VEGF-D protein detected in any UtA branches contra-lateral to the side that had been injected with Ad.VEGF-D^ΔNΔC^. There was no human VEGF-D detectable by ELISA in any UtA, uterine wall, or placentome sample collected from long-term transduced ewes and sham controls. VEGF-D was also not detected in maternal or fetal blood/serum samples obtained at vector injection or at post-mortem examination in short-term and long-term experiments. These findings are similar to our previous findings for Ad.VEGF-A_165_ delivery in the UtAs [14], [15].

**Table 4:** VEGF-D protein detected by ELISA in uterine artery, uterus and placentome samples 4–7 days after injection of Ad.VEGF-D^ΔNΔC^ or Ad.LacZ vectors in two animals.

Sample	VEGF-D protein concentration (pg/mg) on Ad.VEGF-D^ΔNΔC^ injected side (n = 2)	VEGF-D protein concentration (pg/mg) on Ad.LacZ injected side (n = 2)
Uterine artery – Main	Nd; 632.96	Nd, Nd
Uterine artery – 1st branch	335.58; 577.53	Nd, Nd
Uterine artery – 2nd branch	429.61, Nd	Nd, Nd
Uterine artery – 3rd branch	228.46; 269.76	Nd, Nd
Uterus	358.16, Nd	Nd, Nd
Placentome	Nd, Nd	Nd, Nd

Nd: Not detectable.

### eNOS, Akt and Erk levels

**Figure 5:**
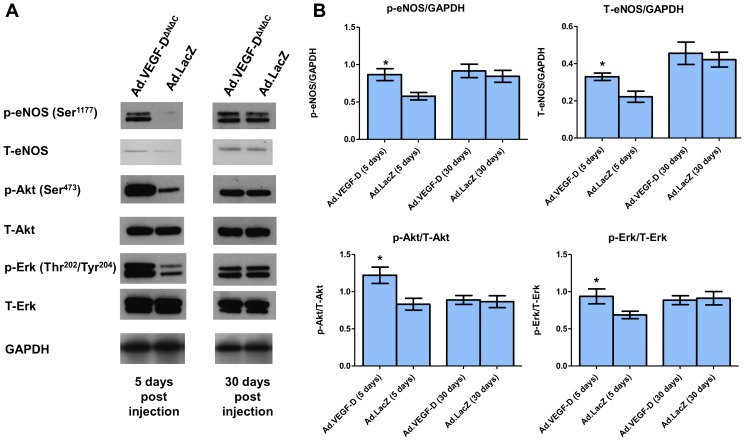
Effects of Ad.VEGF-D^ΔNΔC^ uterine artery transduction on phosphorylated (p) and Total (T) eNOS, Akt and Erk expression. (A) A representative blot shows upregulation of p-eNOS (Ser^1177^), T-eNOS, p-Akt and p-Erk in Ad.VEGF-D^ΔNΔC^ transduced UtAs compared to Ad.LacZ transduced UtAs 5 days after vector administration, but not 30 days after vector administration. Results are representative of n = 3 independent experiments each for the short-term and long-term time points. GAPDH was used as a loading control. (B) Densitometric analysis was performed on the western blots using Image J software, after normalizing against the density of GAPDH, T-Akt or T-Erk, as appropriate. Results are representative of n = 3 independent experiments. * indicates p<0.05 (t-test).

Protein extracts of UtA samples from short-term studies (4–7 days after vector injection) and long-term studies (30–45 days after vector injection) were analysed for changes in phosphorylated and total levels of eNOS, Akt and Erk by western blotting. We observed significantly increased levels of p-eNOS (Ser^1177^), T-eNOS, p-Akt and p-Erk in Ad.VEGF-D^ΔNΔC^ transduced UtAs short-term. However, this difference was not sustained long-term (Figure 5).

### Neovascularization and Endothelial cell proliferation

**Figure 6:**
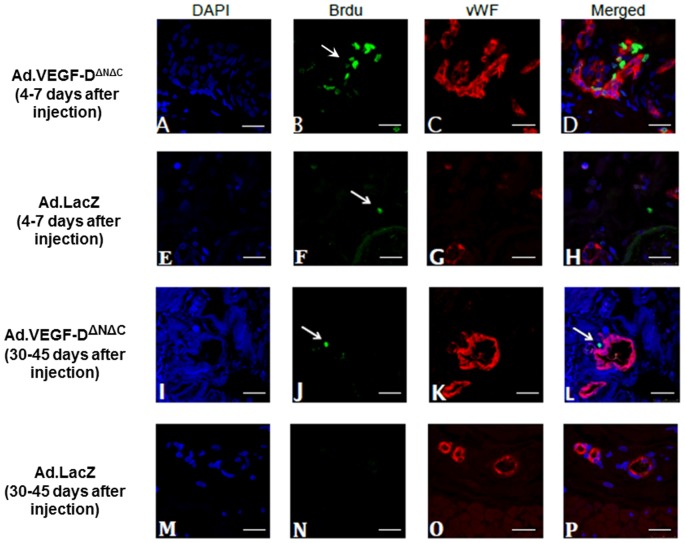
Proliferation and Neovascularization in Ad.VEGF-D^ΔNΔC^–transduced uterine arteries. Clusters of proliferating endothelial cells in the short and long term sheep injected with Ad.VEGF-D^ΔNΔC^ (A–D and I-L respectively) and with Ad.LacZ (E–H and M–P respectively). Pictures A,E,I,M, show the staining of the nuclei with DAPI. The arrows in pictures B,F,J show nuclei which are positive to BrdU. The column containing C,G,K,O pictures shows positive staining to vWF. The merged pictures D,H,L,P, show the positive association of the BrdU stained nuclei with vWF which confirms that these nuclei belong to proliferating endothelial cells. Scale bar = 50 µm.

Four to seven days after transduction we observed a significant increase in the number of proliferating endothelial cells in the main branch of Ad.VEGF-D^ΔNΔC^ transduced UtAs compared to Ad.LacZ transduced UtAs or untransduced UtAs from control sheep at the same gestational age (p = 0.013, n = 4, Two-way ANOVA, Figure 6). ANOVA showed that the vector type had a significant effect on the number of proliferating endothelial cells but whether the UtA was supplying the gravid or non-gravid uterine horn did not (p = 0.563). There was no significant difference in the number of adventitial blood vessels (p = 0.301, n = 4, Two-way ANOVA) between the Ad.VEGF-D^ΔNΔC^ transduced UtAs and Ad.LacZ transduced UtAs. The mean number of proliferating endothelial cells and adventitial blood vessels in the Ad.VEGF-D^ΔNΔC^/Ad.LacZ transduced UtAs and untransduced UtAs is summarized in Table 5.

**Table 5:** Mean number of proliferating endothelial cells and adventitial blood vessels in the uterine arteries of pregnant sheep transduced with Ad.VEGF-D^ΔNΔC^ or Ad.LacZ and from uninjected sheep.

Treatment administered	Duration of experiment	Mean no. of proliferating endothelial cells (±SEM)	Mean no. of adventitial blood vessels (±SEM)
Ad.VEGF-D^ΔNΔC^ (n = 4)	4–7 days	22.83±6.03*	55.10±6.82
Ad.LacZ (n = 4)		9.16±2.68	50.41±5.51
No treatment (n = 2)		7.75±1.89	47.68±5.40
Ad.VEGF-D^ΔNΔC^ (n = 4)	30–45 days	23.47±6.16	77.91±6.76*
Ad.LacZ (n = 4)		15.5±4.37	58.06±5.78
No treatment (n = 2)		7.8±3.74	54.33±7.26

* indicates significantly greater (p<0.05) compared to Ad.LacZ (control) by two-way ANOVA.

After long-term transduction we observed a tendency to higher numbers of proliferating endothelial cells in the Ad.VEGF-D^ΔNΔC^ transduced UtAs compared to Ad.LacZ transduced and uninfected UtAs, though this increase was not significant. (p = 0.159, n = 4, Two-way ANOVA, Table 5). The number of adventitial blood vessels was significantly greater in the Ad.VEGF-D^ΔNΔC^ transduced UtAs compared to Ad.LacZ transduced and uninjected UtAs (p = 0.043, n = 4, Two-way ANOVA). ANOVA showed that whether the uterine artery was supplying a gravid or non-gravid uterine horn had no significant effect on the number of adventitial blood vessels (p = 0.436).

### Vascular permeability and inflammation

**Figure 7:**
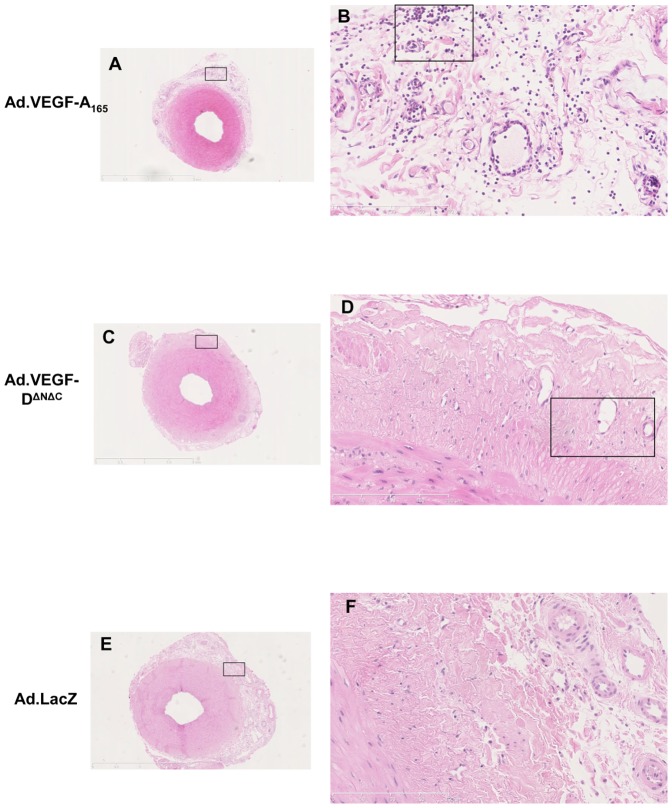
Representative H&E stained pictures of uterine artery sections treated with Ad.VEGF-A_165_ (A,B), Ad.VEGF-D^Δ^ ^NΔC^ (C,D) or Ad.LacZ (E.F). The boxed areas in pictures A, C and E have been magnified in pictures B, D and F respectively. The boxed areas in pictures B and D have been magnified in Figure 8.

**Figure 8:**
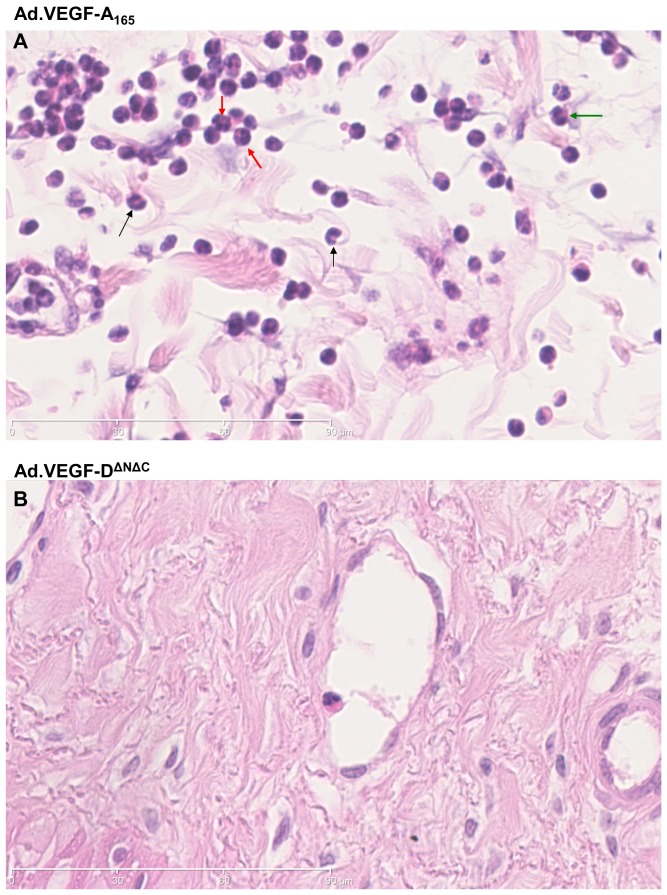
Representative H&E stained pictures of uterine artery sections treated with (A) Ad.VEGF-A_165_ or (B) Ad.VEGF-D^ΔNΔC^. The arrows point towards leucocytes which leak into the adventitia due to enhanced permeability on account of VEGF-A_165_ over-expression. The black arrows point towards monocytes (horse-shoe shaped nuclei); red arrows towards neutrophils (polymorphic nuclei) and green arrow towards basophils (bilobed nuclei).

H&E stained sections of the uterine arteries treated with either Ad.VEGF-A_165_, Ad.VEGF-D^ΔNΔC^ or Ad.LacZ were examined microscopically to look for the presence of inflammatory cells, if any. The adventitia of Ad.VEGF-A_165_ treated vessels appeared more diffuse than that of Ad.VEGF-D^ΔNΔC^ or Ad.LacZ treated vessels, suggestive of edema, and also had a greater number of nucleated cells (Figures 7 and 8). Higher magnification images showed that inflammatory cells, particularly neutrophil polymorphs, monocytes and basophils could be identified in the adventitial layer of Ad.VEGF-A_165_ treated arteries but not Ad.VEGF-D^ΔNΔC^ treated arteries (Figure 8).

### Maternal haemodynamics

**Figure 9:**
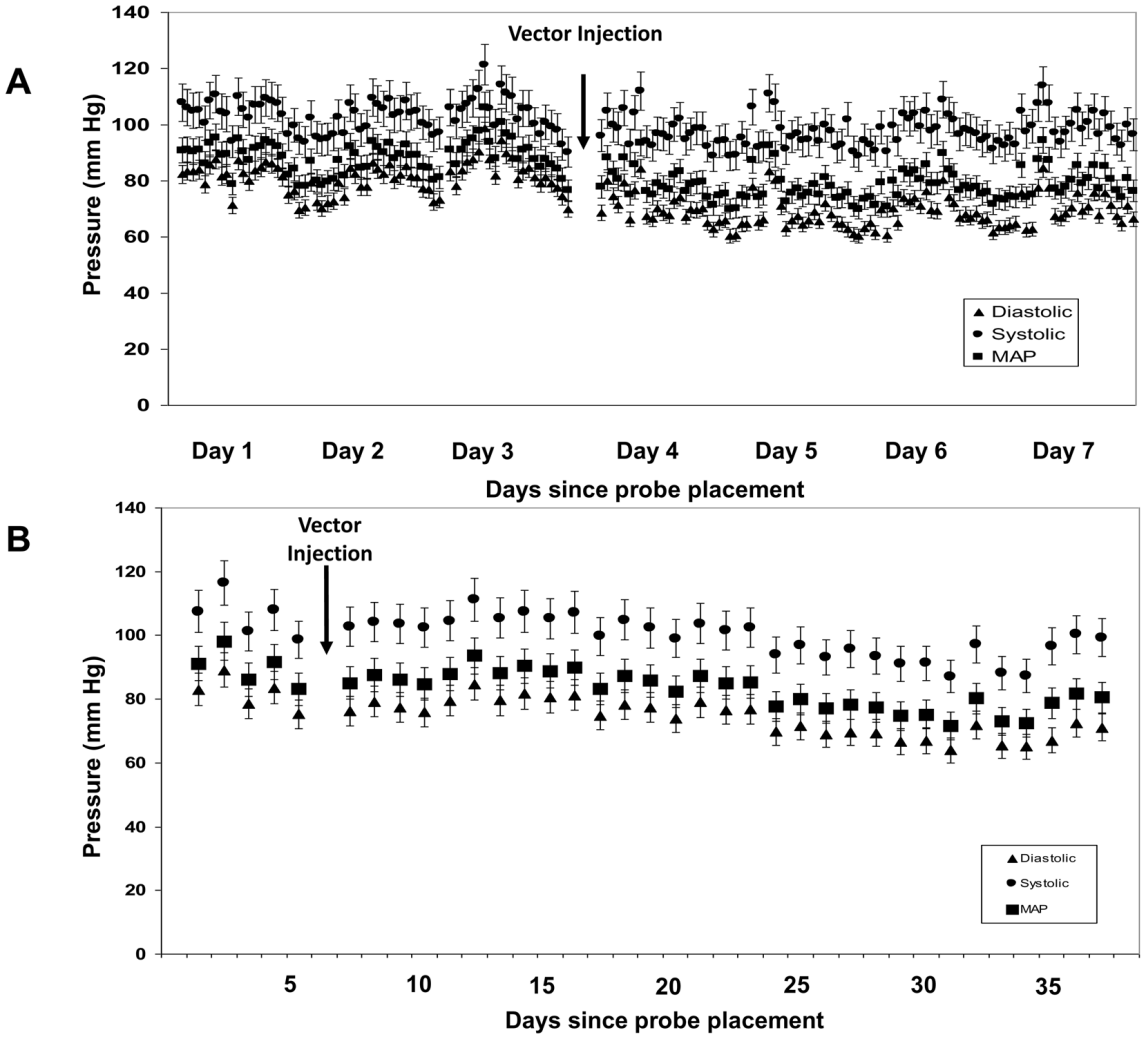
Representative graph showing the (A) diurnal short-term and (B) hourly long-term variation in maternal blood pressure before and after the administration of the vector. There were no significant changes in maternal haemodynamics after Ad.VEGF-D^ΔNΔC^ administration when compared to baseline. Error bars represent SEM.

Maternal blood pressure (BP) was monitored in 5 ewes. There were no short term changes in blood pressure in the first 2 days after vector injection (Figure 9), when VEGF-D^ΔNΔC^ expression would be expected to be at a maximum level. By 7 days after vector injection, the maternal mean arterial pressure had increased marginally from 83.39±2.65 mmHg at baseline to 85.60±8.15 mmHg. This change is similar to our observations in the sham-injected control ewes (85.57 mmHg to 88.13 mmHg).

### UAEC Experiments

**Figure 10:**
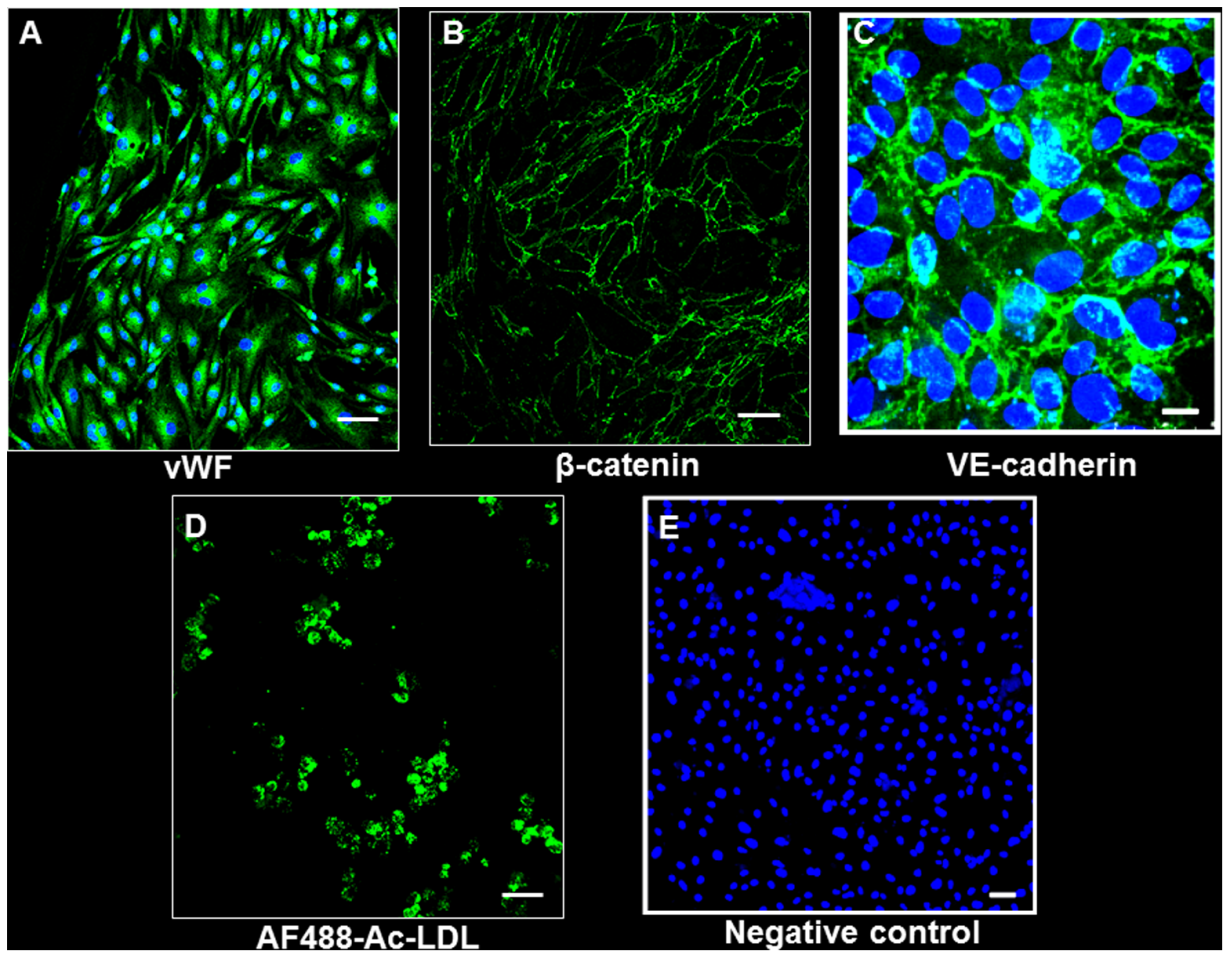
Staining to confirm endothelial identity of pregnant sheep uterine artery endothelial cells (UAECs). Endothelial identity was confirmed by (A) anti-vWF staining; (B and C) cobble-stone shaped appearance following staining with anti-β-catenin and anti-VE-cadherin respectively; (D) uptake of fluorescently labeled Ac-LDL. (E) is a representative negative control wherein the addition of the primary antibody was omitted. Scale bar = 100 µm (A, D and E); 50 µm (B and C).

**Figure 11:**
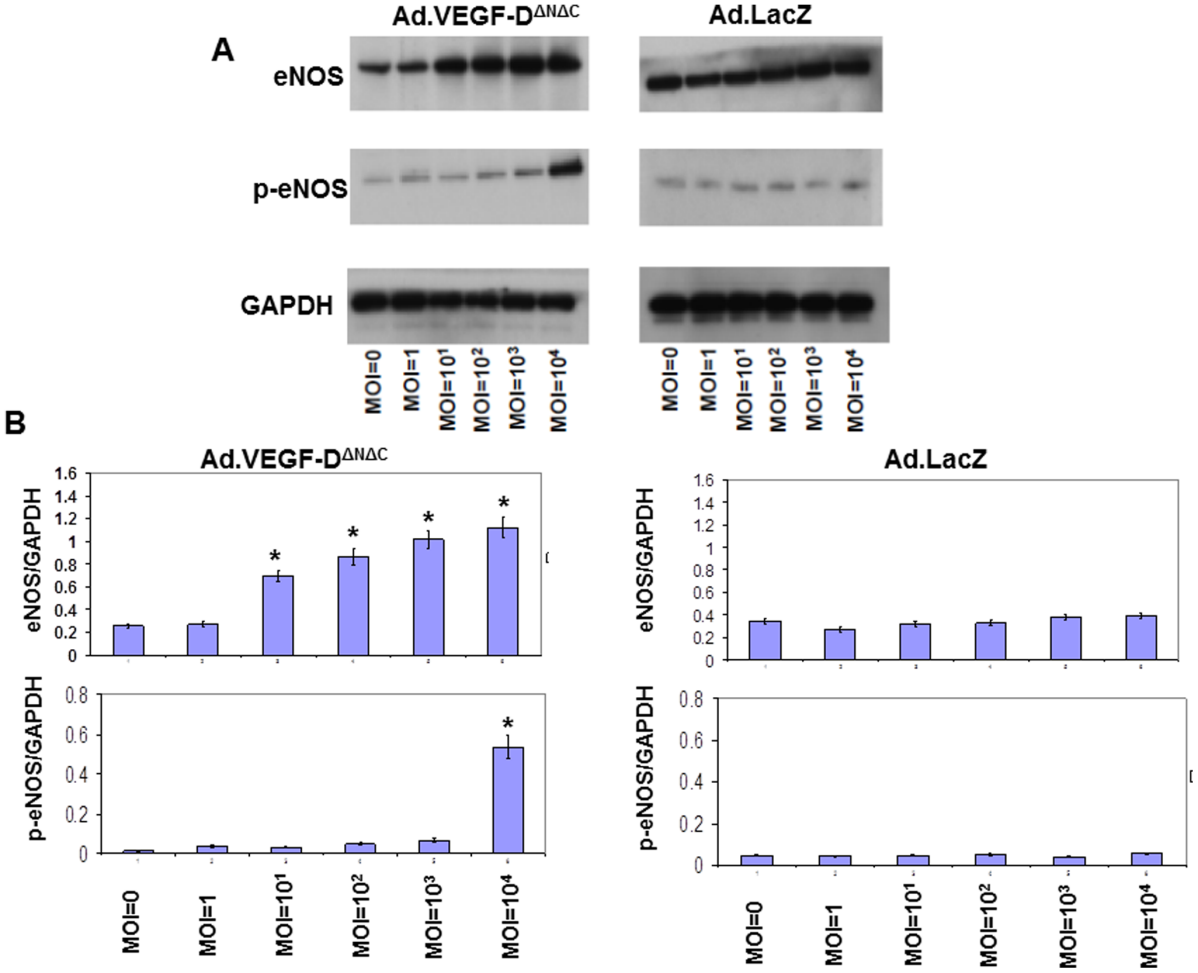
Representative western blots showing an upregulation in eNOS and phospho(p)-eNOS (Ser^1177^) levels 48 hours after Ad.VEGF-D^ΔNΔC^ infection in pregnant sheep UAECs. Pregnant sheep UAECs were grown in culture for up to 4 passages, and then infected at increasing multiplicities of infection (MOIs) with Ad.VEGF-D^ΔNΔC^ or Ad.LacZ in 6-well plates. Protein was extracted from infected cells 48 hours later, and assayed for eNOS and p-eNOS (Ser^1177^) levels by western blotting. (A) An increase in eNOS and p-eNOS (Ser^1177^) levels with increasing MOI was observed in Ad.VEGF-D^ΔNΔC^ infected cells, but not Ad.LacZ infected cells. (B) Densitometric analysis was performed on the eNOS and p-eNOS (Ser^1177^) bands using Image J software, after normalizing against the density of GAPDH bands. Results are representative of n = 3 independent experiments. * indicates p<0.05 in comparison to the relative density of the corresponding band from uninfected cells (MOI = 0) by t-test.

**Figure 12:**
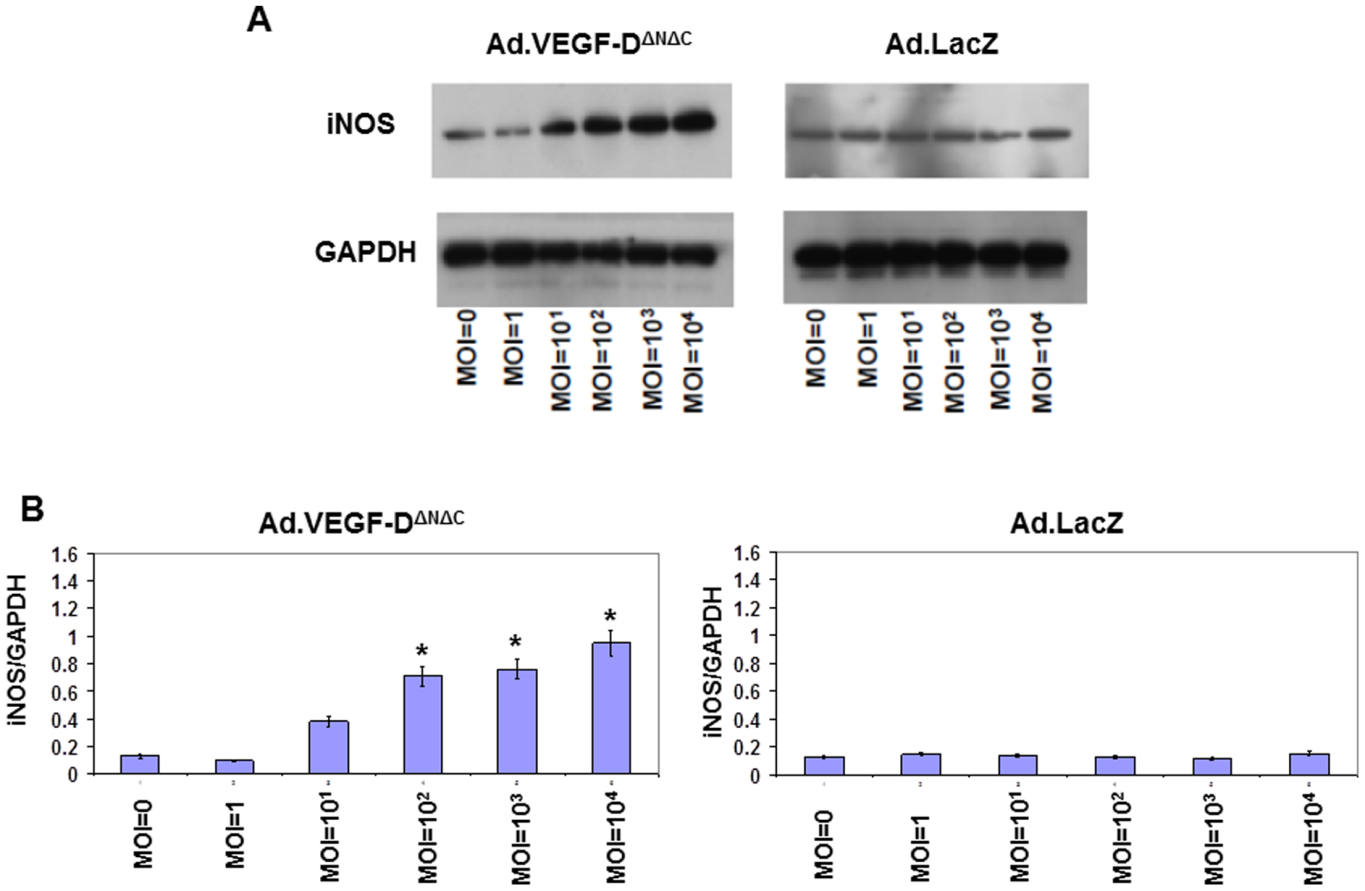
Representative western blots showing an upregulation in iNOS levels 48-D^ΔNΔC^ infection in pregnant sheep UAECs. Pregnant sheep UAECs were grown in culture for upto 4 passages, and then infected at increasing MOIs with Ad.VEGF-D^ΔNΔC^ or Ad.LacZ in 6-well plates. Protein was extracted from infected cells 48 hours later, and assayed for iNOS levels by western blotting. (A) A dramatic increase in iNOS levels with increasing MOI was observed in Ad.VEGF-D^ΔNΔC^ infected cells, but not Ad.LacZ infected cells. (B) Densitometric analysis was performed on the iNOS bands using Image J software, after normalizing against the density of GAPDH bands. Results are representative of n = 3 independent experiments. * indicates p<0.05 in comparison to the relative density of the corresponding band from uninfected cells (MOI = 0) by t-test.

**Figure 13:**
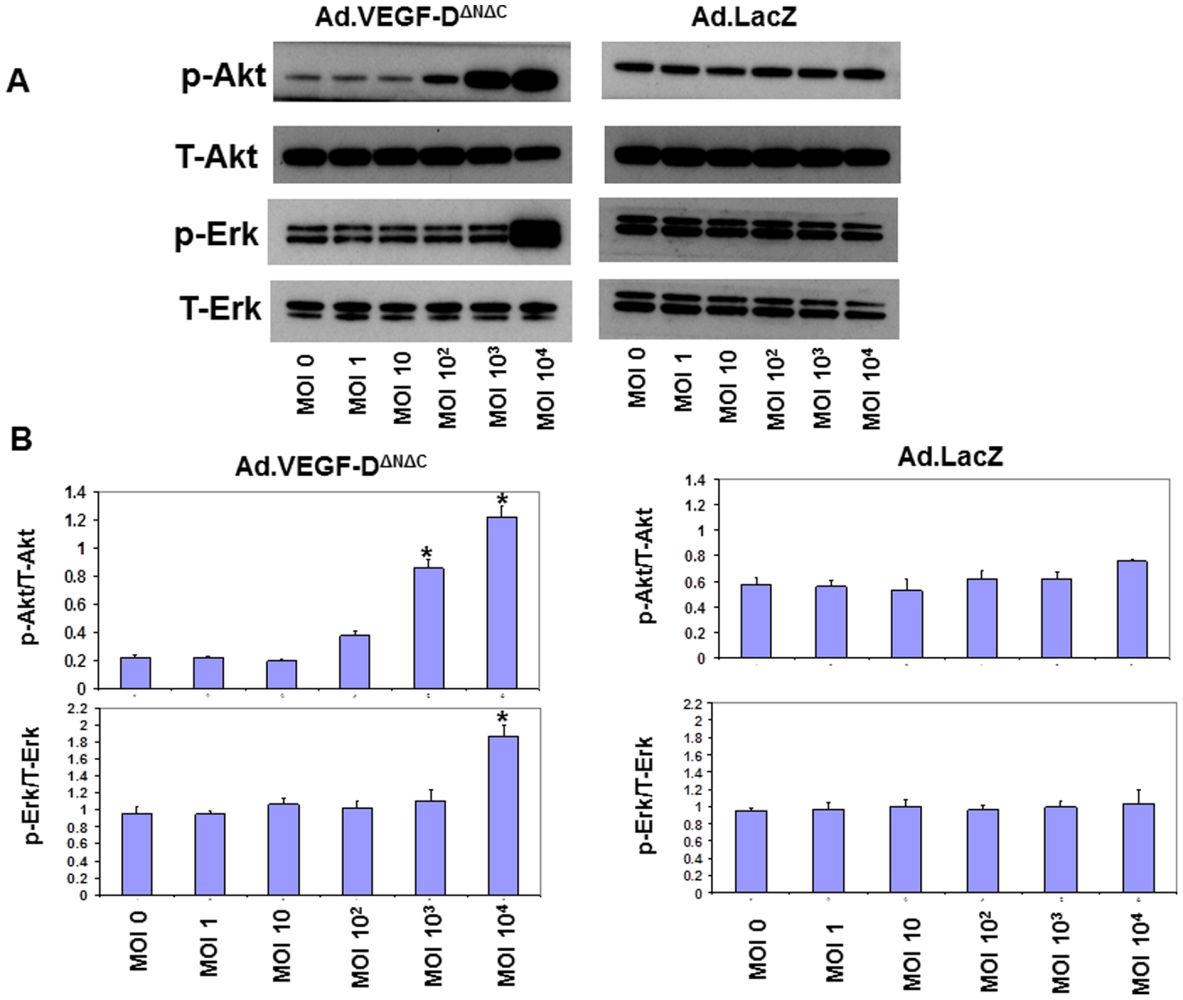
Representative western blots showing an upregulation in p-Akt and p-Erk levels 48 hours after Ad.VEGF-D^ΔNΔC^ infection in pregnant sheep UAECs. Pregnant sheep UAECs were grown in culture for up to 4 passages, and then infected at increasing MOIs with Ad.VEGF-D^ΔNΔC^ or Ad.LacZ in 6-well plates. Protein was extracted from infected cells 48 hours later, and assayed for p-Akt, T-Akt, p-Erk and T-Erk levels by western blotting. (A) An increase in p-Akt and p-Erk levels with increasing MOI was observed in Ad.VEGF-D^ΔNΔC^ infected cells, but not Ad.LacZ infected cells. (B) Densitometric analysis was performed on the p-Akt and p-Erk bands using Image J software, after normalizing against the density of T-Akt and T-Erk bands respectively. Results are representative of n = 3 independent experiments. * indicates p<0.05 in comparison to the relative density of the corresponding band from uninfected cells (MOI = 0) by t-test.

To further investigate the mechanisms mediating the VEGF-D^ΔNΔC^-induced reduction in UtA vasoconstriction, the possibility was examined that adenoviral VEGF-D^ΔNΔC^ over-expression could induce expression of eNOS and/or iNOS in primary cultures of sheep UAECs. Isolated UAECs showed a typical cobblestone morphology, and stained positively with fluorescently tagged Ac-LDL, anti-vWF, anti-VE cadherin and anti β-catenin, confirming their endothelial identity (Figure 10). We observed a significant upregulation in the levels of eNOS, p-eNOS(Ser^1177^) and iNOS 48 hours post-infection in the Ad.VEGF-D^ΔNΔC^ infected cells, compared to Ad.LacZ-infected cells (Figure 11 and Figure 12). While the levels of eNOS and iNOS appeared to increase in a dose dependent manner in response to Ad.VEGF-D^ΔNΔC^ infection, the levels of p-eNOS (Ser^1177^) were significantly raised only at the highest MOI of Ad.VEGF-D^ΔNΔC^. We also examined changes in downstream signaling pathways of VEGF by measuring levels of activated p-Akt and p-Erk, and found that Ad.VEGF-D^ΔNΔC^ infection resulted in a significant increase in the active forms of Akt and Erk compared to Ad.LacZ infection (Figure 13), similar to the effects of short-term adenoviral transduction in vivo.

## Discussion

We have studied the effects of local adenovirus-mediated over-expression of VEGF-D^ΔNΔC^ in the UtAs of pregnant sheep at 4–7 days (short-term) and 30–45 days (long-term) after transduction. Transgenic VEGF-D protein expression was observed in uteroplacental tissues at the short-term but not the long-term time point. We observed that Ad.VEGF-D^ΔNΔC^ transduction is associated with an enhanced relaxation response short-term and a reduction in the contractile response at both the short-term and long-term time points. These changes in vascular reactivity were concomitant with a tendency to increased UABF long-term. The changes in UABF we observed did not reach significance, most probably on account of the limited number of animals used in this study. Nevertheless, the magnitude of the changes observed (∼50% increase in UABF in Ad.VEGF-D^ΔNΔC^ transduced vessels v/s 27% increase in Ad.LacZ transduced vessels at 28 days post-injection) indicates that Ad.VEGF-D^ΔNΔC^ transduction does have effects on UABF. Our findings further suggest that the mechanism of action is due to an upregulation of eNOS and increased endothelial cell proliferation short-term, and adventitial neovascularization long-term.

The results presented in this study suggest that FGR caused by utero-placental vascular insufficiency may potentially be treated by Ad.VEGF-D^ΔNΔC^ gene therapy. VEGF-D^ΔNΔC^ may elicit a more restricted range of biological responses compared with the VEGF-A_165_ isoform, but it is not known to be associated with some of the effects of VEGF-A_165_, such as increased vascular permeability, which are associated with pathophysiology. Although we did not observe any tissue edema (on gross examination) in this or the previous study, histological analysis showed inflammatory infiltration and macrophage margination in association with vascular proliferation in the perivascular adventitia of a few UtAs transduced with Ad.VEGF-A_165_ [14]. VEGF-A_165_ has been shown to profoundly increase vessel permeability leading to extravasation of leucocytes into the surrounding tissue and consequent edema [21]. We did not observe such an effect in the uterine arteries transduced with Ad.VEGF-D^ΔNΔC^. For proper assessment of vascular permeability however, whole animal experiments would need to be performed using intravenous injection of a vital dye like Evans blue, which were not possible to perform in this study. For the experiments described in this study, we used a mature/processed form of VEGF-D designated as VEGF-D^ΔNΔC^. Previous studies from our group have demonstrated that adenoviral vectors encoding the long form of the gene (VEGF-D) had no effects on UABF or UtA vascular reactivity [14].

We observed a short term reduction in UABF for the first few days after vector injection in this study, which was similar to our previous study using Ad.VEGF-A_165_ injection. This decrease was limited to ∼10% and was probably caused by vessel occlusion and consequent trauma during injection. UABF recovered in all treated sheep by day 4 after injection.

Ad.VEGF-D^ΔNΔC^ transduction resulted in a significant reduction in the UtA contractile response at both the short-term and long-term time points, but an enhancement of the relaxation response only at the short-term time point. This is most likely to be because at term, the utero-placental blood vessels are maximally dilated, and VEGF over-expression, which is known to bring about vasodilatation, may be unable to further enhance the relaxation of UtAs. It was further noted that in the short-term treated vessels, the amount of residual relaxation after cumulative inhibition with L-NAME, NS-398 and Apamin was significantly greater in the Ad.VEGF-D^ΔNΔC^ treated vessels compared to Ad.LacZ treated vessels. This may reflect either another as yet unidentified VEGF-mediated relaxation mechanism, or, alternatively, augmentation of NO/EDHF-dependent signaling resulting from the over-expression of VEGF-D^ΔNΔC^. We plan to do further experiments with endothelium-independent vasodilators (like Sodium nitroprusside) to test whether the differences in relaxation observed were indeed mediated by the endothelium.

Ad.VEGF-D^ΔNΔC^ transduction resulted in an upregulation of eNOS, p-eNOS (Ser^1177^), iNOS, p-Akt and p-Erk in pregnant sheep UAECs at the 48 hour time point. Ser^1177^ is the same site phosphorylated in response to shear stress [22] and phosphorylation of this site renders eNOS active at resting Ca^2+^ concentrations. All studies on UAECs were carried out at passage four. Ovine UAECs retain their primary *in vivo* characteristics up to passage four, meaning that expression of key proteins and mRNA are retained. Levels of eNOS protein and mRNA are found to be higher in UAECs from pregnant ewes compared to cells from non-pregnant ewes at the fourth passage [23], [24]. Similar to the findings in UAECs, we observed that Ad.VEGF-D^ΔNΔC^ transduction upregulated eNOS, p-eNOS (Ser^1177^), p-Akt and p-Erk in pregnant UtAs for up to at least 7 days after gene transfer, but this upregulation was no longer evident at the long-term time point. This may be because continued significant VEGF expression is needed for long-term eNOS upregulation or, alternatively, eNOS level at term and in normal pregnancy is already at its peak and cannot be upregulated further by VEGF over-expression. These results suggest that VEGF may be activating eNOS via Phosphoinositide 3-kinase (PI3K) dependent Akt-catalysed phosphorylation. PI3Ks are a family of enzymes which play an important role in cellular physiology, particularly cell growth, proliferation, differentiation, motility, survival and intracellular trafficking. They mediate these functions in response to the binding of different growth factors to cell surface receptors [25].

A significant increase in adventitial endothelial cell proliferation was observed in the UtAs 4–7 days after transduction with Ad.VEGF-D^ΔNΔC^ vector in comparison with control Ad.LacZ vector. The number of proliferating endothelial cells however was not significantly different between the Ad.VEGF-D^ΔNΔC^ and Ad.LacZ transduced UtAs when vessels were analysed at 30–45 days after gene transfer. On the other hand, the number of positively stained anti-vWF blood vessels was significantly greater in the adventitia of UtAs examined 30–45 days after Ad.VEGF–D^ΔNΔC^ transduction but not in vessels examined 4–7 days after adenovirus gene transfer. We speculate that endothelial cells which are stimulated to proliferate by relatively high levels of VEGF-D^ΔNΔC^ at the peak of adenovirus vector expression (2–7 days) subsequently organize themselves into adventitial blood vessels, which results in an increase in perivascular blood vessel number seen in term pregnant UtAs.

Our findings suggest that upregulation of eNOS in the first week after vector injection may be responsible for the initial increase in UABF. The long-term increase in UABF, however, may also be related to enhanced UtA vascularization reflected by an abundant adventitial blood supply. We speculate that these adventitial blood vessels may be the *vasa vasora*. The *vasa vasorum* is a microvascular network that originates primarily in the adventitia of the large arteries and supplies nutrients and oxygen to the outer layers of the arterial wall [26]. Thus, proliferation of the *vasa vasora* may augment the function of the UtA thereby enhancing uterine perfusion. In animal experiments VEGF-A_165_ and VEGF-D^ΔNΔC^ gene transfer is capable of inducing therapeutic angiogenesis in diverse tissues and organ systems. Over-expression of VEGFs using viral vectors stimulates significant neovascularization and supraphysiological increase in perfusion in healthy and ischemic skeletal muscles and myocardium because of increased angiogenesis and capillary enlargement [27]. It is also believed that adventitial microvessels/*vasa vasora* in the utero-placental and ovarian vascular beds play an important role in facilitating the changes in blood flow in pregnancy [28].

One of the limitations of this study was that it was carried out in normal pregnant sheep. During normal pregnancy, the utero-placental vascular bed develops and the vascular channels dilate to facilitate the maximal supply of substrates and oxygen to the developing fetus. It is possible that a larger effect might be observed in pregnancies complicated by FGR related to uteroplacental insufficiency, where there is evidence of reduced trophoblast invasion of the spiral arteries, increased resistance to blood flow and reduced perfusion.

An intravenous infusion of VEGF has been shown to result in transient tachycardia, hypotension and a decrease in cardiac output in conscious instrumented rats [29]. In this study however, we did not observe any changes in maternal haemodynamics, other than a small fall in BP at the end of gestation, which is normally observed in sheep [30]. At the same time, no long-term expression of VEGF-D^ΔNΔC^ could be detected in maternal and fetal tissues by ELISA, which provides re-assurance against long-term toxic effects. In our previous studies, we were only able to detect transgenic VEGF-A_165_ expression by RT-PCR in the uterine arteries that had actually been injected with Ad.VEGF-A_165_, but not in any other maternal or fetal tissue [14], [15]. These findings support our conclusion that UtA injection of Ad vectors results in only local transduction and transgenic protein expression, without eliciting systemic effects which could be deleterious in pregnancy.

This study was designed with clinical relevance in mind. Application of the vector injection technique described in this paper to human patients is particularly challenging. FGR fetuses are frequently hypoxic, and reductions in UABF during UtA occlusion may exacerbate the situation resulting in fetal/neonatal complications. A minimally invasive technique such as transfemoral UtA catheterization with temporary balloon occlusion of the vessel lumen as is used to treat massive obstetric haemorrhage [31] may decrease UtA trauma and post-injection vasoconstriction. Further experiments to determine optimal vector dose and the mode of delivery are required. Another issue is the timing of gene delivery. Fetuses with advanced growth restriction and cardiovascular compensation through brain sparing have a significant degree of hypoxemia but are not acidemic until abnormal precordial venous dopplers are observed that signal decompensation [32]. This should be considered when deciding on the best timing for vector application, and especially that the longitudinal progression of early severe FGR is well defined [33]. In addition, those fetuses with early FGR and very high umbilical artery resistance at presentation (>4SD above the mean for gestation) could be considered at a very high risk for morbidity and mortality [34]. These fetuses would represent the target population for a phase I trial of Ad.VEGF-A_165_/Ad.VEGF-D^ΔNΔC^ gene therapy and application of the vector in this case would be preferable at a stage where significant hypoxemia has not yet developed (i.e. before brain sparing occurs) to prevent acute deterioration during UtA occlusion. Application at this stage would also give time for the increased expression of transgenic VEGF protein to have an effect on UABF and vascular reactivity. If applied too late in the progression of FGR, the delay in any beneficial changes might not prevent irreversible damage to a fetus [35].

## Conclusions

The studies described here demonstrate that local adenovirus-mediated VEGF-D^ΔNΔC^ over-expression in the pregnant sheep UtAs at mid-gestation leads to short and long term changes in UtA vascular reactivity, and a tendency to increased UABF. The mechanism of action is likely due to an upregulation of eNOS and increased endothelial cell proliferation short-term, and adventitial neovascularization long-term. The magnitude of the changes observed in this study in terms of UABF, vascular responses on an organ bath, eNOS upregulation and adventitial neovascularization are similar to those seen after Ad.VEGF-A_165_ injection, without the inflammatory changes that were sometimes observed in the UtA adventitia. VEGF-D^ΔNΔC^ gene therapy has the potential to reverse the impaired uteroplacental perfusion found in the majority of cases of severe early onset FGR. Vector administration appears to be safe, leading to no detrimental changes in maternal haemodynamics or pathology. Studies in growth-restricted small and large animals, optimization of the delivery technique, timing of delivery and further safety evaluation will be required before clinical application could be contemplated.
